# Traumatic Diaphragmatic Hernia or Something Else?

**Published:** 2014-07-10

**Authors:** Suhasini Gazula, Nagarjuna Thakur, Narender Kumar, Prashanth G, Shoukatulla Mohammed

**Affiliations:** 1 Department of Pediatric Surgery, Employees State Insurance Corporation (Esic) Superspeciality Hospital, Sanathnagar, Hyderabad, Andhra Pradesh, India; 2 Department of Anesthesiology, Employees State Insurance Corporation (Esic) Superspeciality Hospital, Sanathnagar, Hyderabad, Andhra Pradesh, India

**Keywords:** Subcutaneous emphysema, Diaphragmatic hernia

## Abstract

Subcutaneous emphysema (SCE) mimicking diaphragmatic hernia on X-ray is hitherto unreported and this case has been presented with the aim to alert the clinician about this unusual presentation of SCE to avert undue panic and initiate appropriate evaluation and management. This case also reemphasizes the importance of a thorough clinical examination, which probably could have revealed the subcutaneous emphysema earlier.

## CASE REPORT

A 1-month-old infant weighing 3500 grams was posted for Lumbosacral meningomyelocoele excision and repair under general anaesthesia. After completing the dural repair, the baby was given Valsalva maneuver to check for cerebrospinal fluid (CSF) leak. A sudden rise in peak airway pressure caused bilateral tension pneumothoraces that were recognized immediately and baby improved with thoracocenteses followed by bilateral intercostal drain insertion. Chest tube position was confirmed with C-arm.

Baby was extubated on table and was shifted to the neonatal surgical intensive care unit. A repeat chest radiograph done next morning showed a well-defined longitudinal gas shadow extending from the left hypochondrium, across the diaphragm into the chest (Fig. 1). The possibility of a diaphragmatic rupture on the left side (following the Valsalva maneuver) with herniation of bowel into the chest was considered. In lieu of barotrauma leading to bilateral tension pneumothorax, the possibility of traumatic diaphragmatic hernia was even more plausible. Clinically however, the baby was asymptomatic with no evidence of decreased air entry or bowel sounds on the left side of the chest on auscultation. Plain CT scan of the chest was done to confirm diagnosis.

**Figure:**
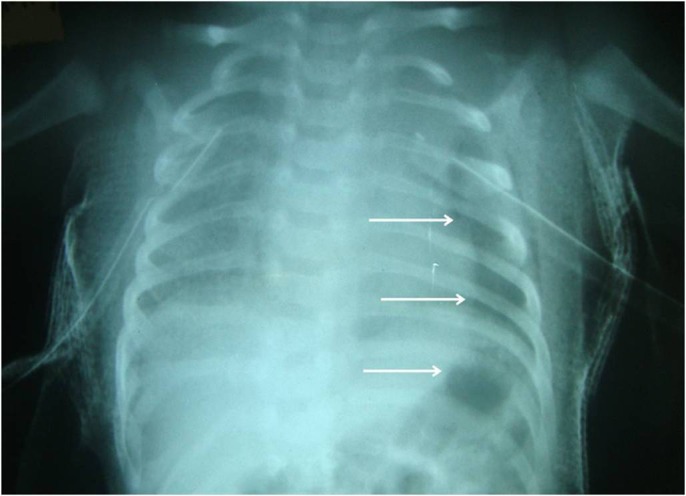
Figure 1: Chest radiograph showing a well-defined longitudinal gas shadow (arrows) extending from the left hypochondrium, across the diaphragm into the chest cavity.

CT-chest showed an intact left hemidiaphragm with no diaphragmatic hernia. However, it showed subcutaneous emphysema extending from D4-D9 spine level on the left posterior thoracic wall (Fig. 2) that was mimicking a bowel gas shadow on the chest radiograph. Retrospectively, a repeat clinical examination revealed a classical crepitus and subcutaneous emphysema over a 3 cm x 4 cm area on the left posterior lower thoracic wall (probably missed as the baby was nursed in the supine position and a bulky chest drain dressing). It resolved spontaneously in 24 hours, the chest drains were removed after 48 hours and baby discharged well.

**Figure:**
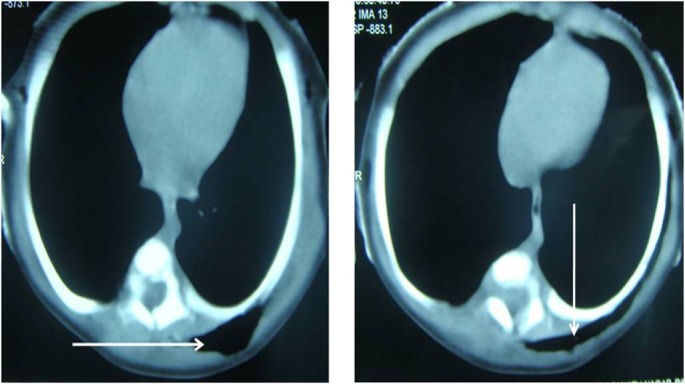
Figure 2: CT scan of the chest showing subcutaneous emphysema (arrows) in the left lower posterior thoracic wall.

## DISCUSSION

Subcutaneous emphysema (SCE), a fairly common clinical finding is categorized as a type of air leak syndrome, the others being pulmonary interstitial emphysema, pneumothorax, pneumomediastinum, pneumopericardium, pneumoperitoneum and systemic air embolism. Neonatal air leaks may present as a thoracic emergency requiring emergent intervention [1]. Emergent needle aspiration and/or tube drainage are necessary in managing tension pneumothorax or pneumopericardium with cardiac tamponade. When the air leak is asymptomatic and the infant is not mechanically ventilated, there is usually no specific treatment [1].

Subcutaneous emphysema can occur as a result of trauma, iatrogenic, infectious causes or even spontaneously. The most common cause of air leak syndrome in neonates is inadequate mechanical ventilation of the fragile and immature lungs and its incidence is inversely related to the birth weight, especially in very-low-birth-weight and meconium-aspirated infants [1]. SCE is also a common occurrence after chest-tube insertion, thoracoscopic and laparoscopic procedures. Rare causes of SCE like gastric perforation [2], necrotizing enterocolitis [3], and fibre-optic endotracheal intubation [4] have also been reported in neonates. To prevent air leak syndrome, gentle ventilation with low pressure, low tidal volume, low inspiratory time, high rate, and judicious use of positive end expiratory pressure are the keys to caring for mechanically ventilated infants [1].

Subcutaneous emphysema has a great importance due to its broad casualty, some causes being totally benign, but others potentially lethal. In addition, SCE can occasionally mimic other conditions like necrotizing fasciitis [5], meningitis [6], cellulitis [7] or diaphragmatic hernia clinically or radiologically leading to confusion, unnecessary interventions, panic or delays in management. 

SCE mimicking diaphragmatic hernia on X-ray is hitherto unreported and this case has been presented with the aim to alert the clinician about this unusual presentation of SCE to avert undue panic and initiate appropriate evaluation and management. This case also reemphasizes the importance of a thorough clinical examination, which probably could have revealed the subcutaneous emphysema earlier.

## Footnotes

**Source of Support:** Nil

**Conflict of Interest:** None

